# Supplementary material for: Splice-Junction-Based Mapping of Alternative Isoforms in the Human Proteome
Source: Cell Rep. Author manuscript; Available in PMC 2020 Jan 15. (PMC6961840; doi:10.1016/j.celrep.2019.11.026)
Supplement: 2 [file NIHMS1546469-supplement-2.zip › DF1/PXD009737/TestisGluC/TestisGluC_1_PARK7_MRRAGGPYDVVVLPGGNLGAQNLSESAAVKE.pdf]

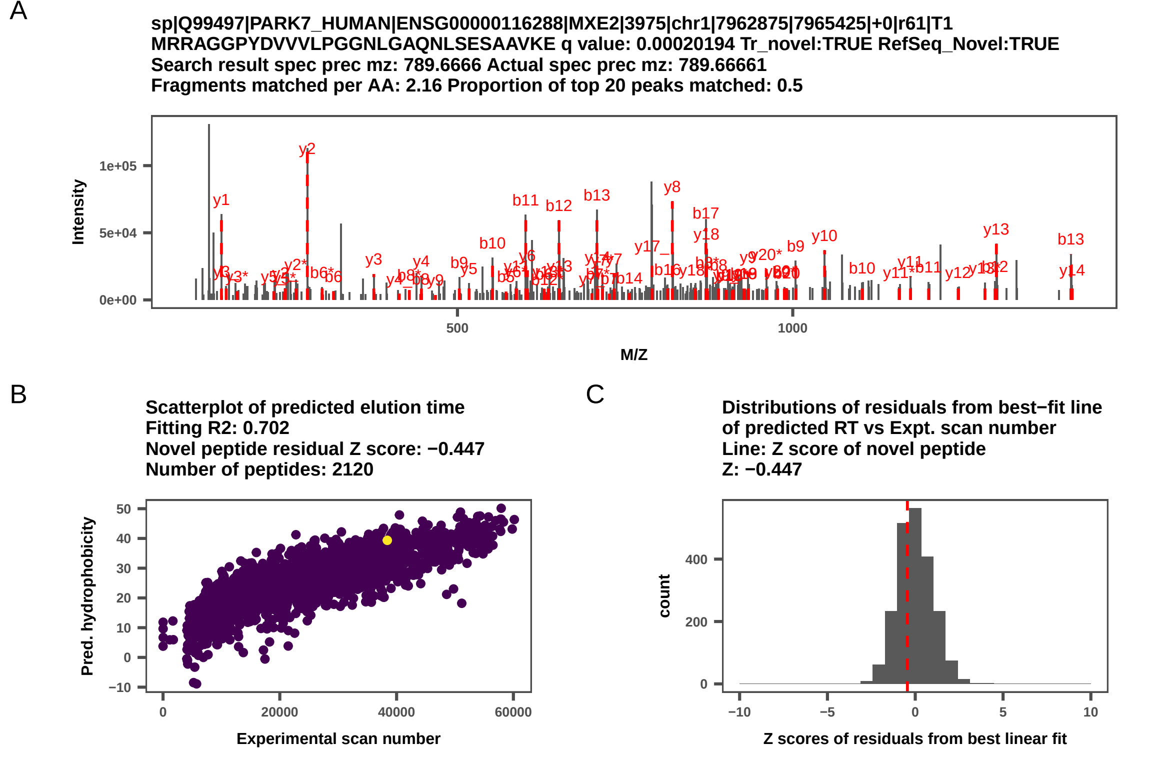
A
sp|Q99497|PARK7_HUMAN|ENSG00000116288|MXE2|3975|chr1|7962875|7965425|+0|r61|T1
MRRAGGPYDVVVLPGGNLGAQNLSESAAVKE q value: 0.00020194 Tr_novel:TRUE RefSeq_Novel:TRUE
Search result spec prec mz: 789.6666 Actual spec prec mz: 789.66661
Fragments matched per AA: 2.16 Proportion of top 20 peaks matched: 0.5
Intensity
1e+05
5e+04
0e+00
500
1000
M/Z
y1
y2
b11
b12
b13
y8
b17
y18
b10
y10
y13
b13
b9
y14
B
Scatterplot of predicted elution time
Fitting R2: 0.702
Novel peptide residual Z score: −0.447
Number of peptides: 2120
Pred. hydrophobicity
50
40
30
20
10
0
−10
0
20000
40000
60000
Experimental scan number
C
Distributions of residuals from best−fit line
of predicted RT vs Expt. scan number
Line: Z score of novel peptide
Z: −0.447
count
400
200
0
−10
−5
0
5
10
Z scores of residuals from best linear fit
